# Antiepidermal Growth Factor Receptor Therapy in Squamous Cell Carcinoma of the Head and Neck

**DOI:** 10.1155/2012/521215

**Published:** 2012-06-19

**Authors:** Walid Shaib, Scott Kono, Nabil Saba

**Affiliations:** Winship Cancer Institute, Emory University, 1365 Clifton Road Northeast Atlanta, GA 30322, USA

## Abstract

Squamous cell carcinoma of head and neck (SCCHN) is the most common neoplasm of the upper aerodigestive tract. In this paper, we attempt to summarize the role and applications of the epidermal growth factor receptor (EGFR) inhibitors monoclonal antibodies (moAbs) and tyrosine kinase inhibitors (TKIs) locally advanced as well as metastatic SCCHN. Targeted therapy in SCCHN is now incorporated in the first-line regimes for advanced disease. Novel targeted agents, including the EGFR antibody, cetuximab, have been approved for use as single agents or in combination with radiation therapy or chemotherapy in treatment of recurrent metastatic or locally advanced SCCHN. Refractory mechanisms that bypass the pathway of EGFR inhibitors activity are identified explaining resistance to targeted therapy. Strategies of cotargeting EGFR and other pathways are under investigation. Examples of targeted therapy being used include mammalian target of rapamycin (mtor) inhibitors, antivascular endothelial growth factor (VEGF) moAb, and other inhibitors. We will be focusing our paper on the preclinical and clinical aspects of EGFR inhibition in SCCHN and touch upon other targeted therapies in application.

## 1. Introduction

It is estimated that about 49,260 new cases of the oral cavity, pharyngeal and laryngeal cancers and 11,480 cancer deaths occurred in 2010 [1]. Squamous cell carcinoma accounts for more than 90% of head and neck cancers. EGFR is expressed in normal tissues including the gastrointestinal tract, dermis, and kidneys. An overexpression of the receptor or any of its linked pathways occurs in most epithelial cancers and in 90% of SCCHN. EGFR expression in SCCHN is 1.7-fold than that in normal cells (*P* = 0.005) [2, 3]. EGFR overexpression is an early event in SCCHN carcinogenesis; it is detected in “healthy” mucosa of cancer patients more often than healthy controls and its expression increases steadily with different grades of premalignancies from hyperplasia to low-grade and high-grade dysplasia to invasive carcinoma [4]. High levels of EGFR are correlated with poor prognosis and resistance to radiation therapy in a variety of cancers including SCCHN [5]. Genomic profiles were identified as predictors of radiation-resistant SCCHN [6]. Moreover, development of the rash is likely mechanistically related to inhibition of the EGFR and has been associated in several individual studies with better outcomes [7].

Given this, EGFR has its protumor effect and blockade of its pathways has been investigated as a rational anticancer strategy in different malignancies including SCCHN [8]. Monoclonal antibodies to EGFR, Cetuximab, Panitumumab, and Zalutumumab, have been the most investigated in SCCHN. In addition, low molecular weight tyrosine kinase inhibitors (TKIs) including Gefitinib (Iressa; AstraZeneca, Wilmington, Del) and Erlotinib (Tarceva; OSI Pharmaceuticals, Melville, NY/Genentech, South San Francisco, Calif). Newer “dual TKIs” that inhibit both EGFR and HER-2 have also been investigated.

## 2. Epidermal Growth Factor Receptor ****(EGFR) Action Pathway

**Figure 1:**
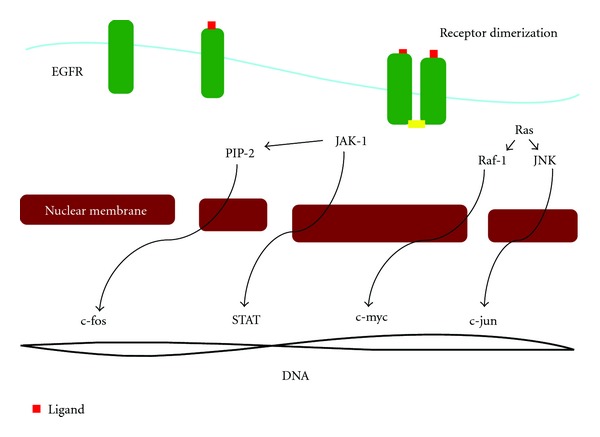
Schema of the EGFR activation pathway. EGFR is the transmembrane protein (green) with intrinsic tyrosine kinase (phosphorylation in yellow) activity that regulates cell growth. Its ligands are EGF, tumor necrosis factor alfa (TNF*α*) and others (red). Ligand binds to the receptor and initiates the activity of signaling pathways through dimerization of the receptor and autophosphorization of the tyrosine residues in the cytoplasm, which activates other downstream pathways including Janus Kinase (JAK) Signal Transducers and Activators of Transcription (STAT) directly and through phosphatidylinositol 4,5 bisphosphate (PIP2), c-Jun N-Terminal Kinase (JNK) and raf-1. Activation of these pathways leads to activation of STAT, c-Fos, c-Jun, and c-myc transcription factors respectively. These transcription factors regulate gene expression leading to cell cycle progression, proliferation, invasion, angiogenesis and metastasis.

The EGFR is the cell-surface receptor for members of the epidermal growth factor (EGF) family of extracellular protein ligands. It is a member of the ErbB family of receptors, a subfamily of four closely related receptor tyrosine kinases: ErbB-1, Her 1, Her 2, Her 3, and Her 4 [9]. EGFR is a glycoprotein of 170 kDa, encoded by a gene located on chromosome 7p12. Its known ligands are EGF, TGF *α*, amphiregulin, heparin-binding EGF, betacellulin, epiregulin, and NRG2-*α* [10]. EGFR dimerization stimulates its intrinsic intracellular protein-tyrosine kinase activity. As a result, autophosphorylation of several tyrosine residues in the C-terminal domain of EGFR occurs. This autophosphorylation elicits downstream activation and signaling by several other proteins that associate with the phosphorylated tyrosines through their own phosphotyrosine-binding SH2 domains. These downstream signaling proteins initiate several signal transduction cascades, principally the MAPK, Akt, and JNK pathways, leading to DNA synthesis and cell proliferation, decreasing apoptosis potential and increasing angiogenesis [11, 12]. Inhibition of the EGFR can affect the extracellular or intracellular domains. Two complementary therapeutic strategies have been developed. Inhibition of the extracellular domain of the receptor with MoAbs prevents activation of the receptor by endogeneous ligands through competitive inhibition; it also results in internalization and degradation of the antibody-receptor complex, downregulating EGFR expression. Targeting the intracellular domain of the receptor with low molecular weight TKIs competes with adenosine triphosphate (ATP) for its binding site on the intracellular domain of EGFR [13].  Figure 1 summarizes the EGFR action pathway.

## 3. HPV and Its Relation to EGFR Expression

 Human Papilloma Virus (HPV-) associated oral cavity and oropharyngeal cancers estimate 1700 new cases in women and 5700 in men annually in the United States [14]. It is not clear why the oropharynx is more susceptible to HPV transformation, although its similarity to the uterine cervix, in terms of easy access for infection and the same embryonic development from endoderm, has been suggested. The mode of transmission of HPV to the oral cavity is less understood and less defined at this stage, but sexual behavior and practices represent possible modes of transmission [15]. In a cohort analyzing 271 tissue samples collected from 1984 to 2004 checking for HPV prevalence in SCCHN concluded that there is an increased prevalence of HPV-positive SCCHN over that period from 16.3% during the 1980s to 72.7% during the 2000s. More importantly, significant improvement in survival over time arise from the long-term survival advantage of HPV-positive SCCHN patients [16]. Furthermore, HPV-positive SCCHN patients have risk factors related to sexual behavior compared to HPV-negative cancers that are strongly associated with tobacco and alcohol use [17]. HPV-related oropharyngeal cancer is now a well-defined entity with well-known characteristics that include young age, good performance status, male gender, nonsmoking and nondrinking status, basaloid tumor histology, and high-risk sexual behavior.

HPV viral oncogenes E6 and E7 are frequently overexpressed in the oropharynx. In a case-control study, D'Souza et al. reported that oropharyngeal cancer was significantly associated with the presence of oral HPV-16 infection. HPV DNA was detected in 72% of 100 oropharyngeal tumor specimens, and 64% of the patients in the study were seropositive for HPV-16 E6, HPV-16 E7, or both [18]. The p16 protein inhibits cdk4- and cdk6-cyclin D functionality. P16 suppresses the hyperphosphorylation of retinoblastoma (Rb), thus inactivating cell proliferation pathways. Rb acts as a negative regulator of p16 expression. One of the critical targets of the G1-specific cdk complexes is the release of E2F through phosphorylation of the pRb-E2F protein complex which is inhibited by p16 [19, 20]. The viral oncoprotein E7 inactivates the RB protein leading to highly increased p16 expression in HPV-positive squamous cell carcinoma [21].

It is unclear how HPV affects the expression of EGFR. Remeirs et al. examined the combination of some of the predictive markers for SCCHN, including HPV-DNA detection, p16, and EGFR expression, in a series of 106 patients diagnosed with SCCHN. P16 overexpression was significantly associated with poorer differentiation of the tumor. Of the p16-positive cases, 19 (65.5%) were poorly differentiated, whereas of the p16-negative cases 33% was poor differentiated (*P*: 0.009). Results also showed a trend toward an inverse correlation of the p16 and EGFR expression that is, p16-positive SCCHN showed less EGFR expression although this did not reach statistical significance (*P*: 0.083). For patients with EGFR+/p16− tumors, the 5-year disease-free survival rate was 39%, while the survival rate of patients with EGFR−/p16+ tumors was 93% (*P*: 0.003). The 5-year overall survival rate of EGFR+/p16− tumors was 38%, compared with the EGFR−/p16+ group, which showed a significantly better outcome of 79%, (*P*: 0.010). Twenty eight percent of the tumors was HPV positive [22]. Reported interaction of HPV proteins with EGFR has shown that HPV-E5 expression leads to elevated EGFR expression in cell culture models [23]. Similar to Remiers et al. findings, Kumar et al. studied the correlations of HPV-DNA, p16, EGFR, and other variables, it was found that EGFR expression was inversely associated with response to induction chemotherapy (*P*: 0.01), chemotherapy/radiotherapy (*P*: 0.055), overall survival (*P*: 0.001), and disease-specific survival (*P*: 0.002) and was directly associated with current smoking (*P*: 0.04), female sex (*P*: 0.053), and lower HPV titer (*P*: 0.03) [24]. Kim et al. reported an association between HPV and p16 expression and an inverse relationship between HPV and EGFR expression but did not report any association with outcome in a series of patients with tonsillar cancer (73% HPV positive) [25]. In a study by Rischin et al., 97 patients were checked for p16 immunohistochemistry stain and EGFR status by fluorescence in-situ hybridization (FISH) to find a correlation between theses 2 variables. EGFR FISH positive tumors consist of 22% (*n* = 97) and p16 was positive in 41% of the cohort. Only 1/97 tumors was positive for both markers. EGFR FISH positivity was associated with inferior failure free survival (HR 2.8, *P*: 0.022) and OS (HR 2.3, *P*: 0.057). This also indicates that EGFR positivity is inversely proportional to p16 expression in SCCHN [26]. In a phase III trial where cetuximab was given concurrently with radiation, Bonner et al. evaluated the efficacy of this regimen and noted an improvement in survival in patients who are younger. The HPV status in this study was not checked but the proposed improvement could be related to HPV itself as it is more prevalent in the young population with predominant oropharynx cancers [27].

Recently the eastern cooperative Oncology group (ECOG) has completed a phase II clinical trial using induction chemotherapy with cisplatin, Paclitaxel, and Cetuximab for HPV-/p16- positive SCCHN with subsequent stratifications to different doses of radiation therapy based on clinical response. The trial accrued close to 80 patients, and the results are being analyzed.

The Radiation Therapy Oncology group (RTOG) recently initiated a clinical trial for the same patient population randomizing patients to Cisplatin and Radiation versus Cetuximab and Radiation therapy (RTOG 1016). The results of both these trials will shape our future approach to clinical management of this patient population and better determine the role of EGFR monoclonal antibodies in treating this disease.

## 4. Preclinical Studies of Monoclonal ****Abs to EGFR and Tyrosine Kinase Inhibitors

MoAb against the extracellular domain of the EGF receptor (moAblO8) concentrates in subcutaneous xenografts of human tumor cells in nude mice and retard cellular growth [28]. The potentiated effect of moABl08 as an antiproliferative agent was shown to be more effective when added to various antineoplastic drugs with the anti-EGF-receptor antibodies. moAbs against the EGF receptor (moAblO8) were found to inhibit or delay the growth of human epidermoid carcinoma cells as xenografts in nude mice. MoAb-mediated antitumor effect could conceivably be enhanced by conjugating the moAbs to doxorubicin, a drug widely used in solid-tumor chemotherapy despite its high toxicity [29].

CP-358,774 (a potent, selective tyrosine kinase inhibitor of human EGFR, produces cell cycle arrest, and initiates apoptosis in human tumor cells overexpressing EGFR) had an antitumor effect in the EGFR-overexpressing human HN5 and human A431 epidermoid carcinomas. Both tumor types are inhibited by specific anti-EGFR antibodies cells that are cultured in xenograft models. Oral administration of CP-358,774 had a significant dose-related antitumor effects against HN5 (EGFR-expressing cells) growing in mice with no thymus gland. Antitumor effects were observed (65–75% inhibition) whether the cytoreductive (cisplatin) agent was dosed before CP-358,774, concurrently with CP-358,774, or after CP-358,774 that is, independent of the sequence of the drug given in relation to cisplatin. In this study, it was shown that there is an additive effect when combining the cytoreductive agent to EGFR inhibitor with greater antitumor effect over a more extended period of time than did the cytoreductive agent alone. CP-358,774 can be administered at the same time with cisplatin with no antagonism in drug action for the cytoreductive agent in this model. The antitumor effects of CP-358,774 correlated well with inhibition EGFR phosphotyrosine in tumor cells [30].

Maurizi et al. retrospectively observed 140 primary with laryngeal squamous cell carcinomas patients and correlated the EGFR status survival. The 5-year survival was 81% for patients with EGFR−tumors compared with 25% for patients with EGFR+ tumors (*P* < 0.0001). The 5-year relapse-free survival was 77% for patients with EGFR−tumors compared with 24% for patients with EGFR+ tumors (*P* < 0.010) [5]. In a phase II study involving 268 patients, EGFR expression was a strong independent prognostic indicator for overall Survival (OS) (*P* = 0.006) and disease free survival (DFS) (*P* = 0.003) and a robust predictor for locoregional relapse but not for distant relapse (*P* = 0.5). The data suggest that EGFR status should be considered for selecting patients for more aggressive combined therapies or enrollment into trials targeting EGFR signaling pathways [31].

## 5. EGFR Inhibitors

Cetuximab is a human murine MoAb of the immunoglobulin G1 (IgG1) isotype that appears to act through multiple mechanisms, and, as an anti-EGFR MoAb, cetuximab blocks the binding of natural ligands to the EGFR, preventing EGFR dimerization, internalization, and autophosphorylation and inhibiting subsequent activation of tyrosine-kinase-mediated signaling pathways. Preclinical studies demonstrated that cetuximab inhibited the growth of EGFR-expressing carcinoma cell lines through cell cycle arrest, antiangiogenesis, antiapoptosis, and inhibition of tumor cell invasion and metastasis. More recent data demonstrated that cetuximab may block the nuclear import of EGFR, preventing activation of DNA repair mechanisms that protect cells from radiation- or chemotherapy-induced DNA damage [32–34].

As the preclinical data were developing in support of combining EGFR inhibitors with radiation, a phase I trial of cetuximab in combination with radiation in patients with advanced head and neck cancer was initiated at the University of Alabama Birmingham. This study of 16 patients provided a potent signal of clinical activity, with 13 of the 15 assessable patients achieving complete response and 2 achieving partial response [35]. These preliminary clinical results, complemented by strong preclinical data, prompted design of a large-scale trial to examine the combination of cetuximab with radiation in advanced head and neck cancer patients that will be reviewed in details below.

In an open, randomized, multicenter, phase I/II study to investigate the safety and tolerability of cetuximab in the first-line treatment of recurrent/metastatic squamous cell carcinoma of the head and neck (SCCHN). The trial enrolled 53 patients and the incidence of dose-limiting toxicities in phase A was acceptable. The most common grade 3/4 adverse events in both groups were leucopenia (38%), asthenia (25%), vomiting (14%), and thrombocytopenia (15%), which are consistent with the known safety profiles of cetuximab, cisplatin/carboplatin, and FU. The overall response rate among patients was 36% [36]. Another phase 1 study evaluating the pharmacokinetic and pharmacodynamic effects of cetuximab and enrolling 39 patients with epithelial malignancies, rash was noted in 26 (67%) patients. Three patients (two with colon cancer and one with laryngeal cancer) achieved a partial response and 13 patients had stable disease. Pharmacokinetic data revealed mean maximum observed cetuximab concentrations and mean area under the concentration-time curve from time zero to infinity increased in a dose-dependent manner up to 400 mg/m^2^ cetuximab. Mean clearance was similar at cetuximab doses ≥100 mg/m^2^, supporting saturation of EGFR binding at 250 mg/m^2^. Pharmacodynamic evaluation revealed that patients with partial response/stable disease had a higher grade rash and higher cetuximab trough levels than those with progressive disease (*P*: 0.032 and 0.002, resp.) [37]. Shin et al. conducted a phase Ib study with cetuximab in combination with cisplatin in patients with recurrent SCCHN. Total of 12 patients who had high levels of EGFR expression and tumors easily accessible for repeated biopsies (pretherapy, 24 hours after first infusion, 24 hours before third infusion) were randomized at three different dose levels of cetuximab (100 mg/m² as a loading dose with maintenance doses at 100 mg/m² weekly; 500 mg/m² as a loading dose with maintenance doses at 250 mg/m² weekly; 400 mg/m² dose as a loading with maintenance doses at 250 mg/m² weekly). High percentage of saturation of EGFR in tumor tissue was maximally achieved at the loading dose of 400 mg/m² followed by a maintenance dose of 250 mg/m² [38].

In a multicenter phase II trial conducted by Vermorken et al. selecting 103 patients with disease progression after receiving 2 to 6 cycles of platinum-based therapy, cetuximab as monotherapy was shown to have a 13% response rate, 46% disease control rate, and 70 days median time to progression. The response to the single agent cetuximab was equivalent to cetuximab added to platinum therapy in platinum-resistant SCCHN [39]. Hitt et al. treated 46 patients with weekly paclitaxel 80 mg/m² and cetuximab. This regimen was also well tolerated. The overall response rate in the 42 evaluable patients was 60%. Median PFS was 5.6 months [40].

### 5.1. EGFR Monoclonal Antibodies

#### 5.1.1. Cetuximab with XRT in Locally Advanced Disease

Bonner et al. conducted a study including 424 with loco-regional stage III or IV advanced head and neck cancer patients who were randomized to treatment with high-dose radiotherapy alone (213 patients) or high-dose radiotherapy plus weekly cetuximab (211 patients). The median duration of locoregional control was 24.4 months among patients treated with cetuximab plus radiotherapy versus 14.9 months in the radiotherapy alone group (*P*: 0.005). The median duration of overall survival was 49.0 months among patients treated with combined therapy versus 29.3 months with the radiotherapy alone group (*P*: 0.03). Progression free survival was also prolonged in the combination arm. The addition of cetuximab to high-dose radiotherapy resulted in a 32 percent reduction in the risk of locoregional progression [27]. This trial supported the use of Cetuximab in concurrence with radiation therapy and lead to its FDA approval for this indication. This combination should not however, be considered as the standard of care as it has not been clearly proven to be equivalent to radiation and platinum-based chemotherapy. Such a randomization is currently being tested on RTOG 1016: however, this includes patients with HPV-/p16-positive disease only.

A recent phase III trial the intrem results of which were presented ASCO 2010, aimed at investigation the addition of cetuximab to patient received 2 cycles of cisplatin concurrently with radiation in a randomized trial RTOG 0522. Eight hundred and ninety five patients are eligible and 447 randomized to addition of cetuximab and 448 to no cetuximab arms. Over 90% of patients received 2 cisplatin cycles in both arms and 74% of cases received the loading plus 6 or more doses of cetuximab in patient receiving EGFR inhibitor for a median follow-up of 2.4 years in surviving patients. The primary endpoint failed to show any significant differences in progression-free survival (*P*: 0.66; 2-year rates: 63% versus 64%), or in overall survival (*P*: 0.17; 2-year rates: 83% versus 80%), with similar death rates within 30 days of therapy in the 2 arm (2.0% versus 1.8%, *P*: 0.81) [41]. Another trial by RTOG is examining the role of adding Cetuximab to radiation therapy in the adjuvant setting for patients with intermediate risks of relapse (RTOG 0920). This trial is currently enrolling.

#### 5.1.2. Cetuximab in Recurrent Metastatic Disease

Cetuximab has been used in combination with different chemotherapeutic agents in recurrent metastatic SCCHN. As a single agent, cetuximab has produced responses that do not exceed 13%. In a study by Herbst et al., cisplatin-unresponsive or minimally responsive disease was assigned to treatment with cisplatin in combination with cetuximab. Partial responses (PRs) were observed in 13% of patients. The highest disease control rate was observed in patients included in the analysis who had stable disease on cisplatin-based therapy [42]. Rozzi et al. combined cetuximab with weekly paclitaxel 80 mg/m² and carboplatin AUC 2 for 3 weeks out of 4 in 33 patients who relapsed after prior platinum-based therapy for their localized disease. The regimen was well tolerated. Response was 45.5% [43]. In a phase II trial evaluating the efficacy and safety of cetuximab, 96 patients were enrolled eligible assessing the response rate of cetuximab added to cisplatin or carboplatin in patients who progressed on platinum based chemotherapy. The response rate, in the intent-to-treat population, was 10%, with a disease control rate (complete response, PR, and stable disease) of 53%. The median time to progression and overall survival were 85 and 183 days, respectively; both were longest in patients achieving a PR (median, 203.5 and 294 days, resp.) [44]. Burtness et al. also reported improved PFS with the addition of cetuximab to cisplatin in ECOG 5397. Patients with recurrent/metastatic squamous cell carcinoma of the head and neck were randomly assigned to receive cisplatin every 4 weeks, with weekly cetuximab or placebo. There were 117 analyzable patients enrolled. Median PFS was 2.7 months for the control arm compared with 4.2 months for the cetuximab arm. Median overall survival showed no difference between the 2 arms. (8 months for versus 9.2 months in the study group *P*: 0.21). Objective response rate was, however, better in the cetuximab group (26% compared to 10% *P*: 0.03). Skin toxicity manifesting as rash was correlated with improved survival (*P*: 0.1) [45]. In a phase III trial by Vermorken et al. (EXTREME study), 442 patients with recurrent or metastatic head and neck cancer were assigned to a first-line regimen of platinum (cisplatin or carboplatin) plus infusional fluorouracil every three weeks with or without cetuximab. Overall, 39% of patients had received chemotherapy at least six months prior to randomization as part of first-line therapy. On the trial, chemotherapy was given for a maximum of six cycles, although cetuximab could be continued as maintenance until disease progression or toxicity. Crossover was not allowed. Chemotherapy plus cetuximab significantly prolonged overall survival compared with chemotherapy alone (median 10.1 versus 7.4 months, hazard ratio for death 0.80, 95% CI 0.64–0.99). Significant improvements in progression-free survival and objective response rates (median 5.6 versus 3.3 months and 36 versus 20 percent, resp.) were also observed. Even though EGFR expression was not a criterion for eligibility, but 83% of patients had tumors with >40% EGFR-positive cells. Importantly, the overall incidence of grade 3/4 toxicities was comparable between the groups except for higher rates of skin toxicity (9% versus <1%, *P*: 0.01), hypomagnesemia (5% versus 1%, *P*: 0.05), and sepsis (4% versus <1%, *P*: 0.02) in the cetuximab group [46]. In a questionnaire based study to evaluate the quality of life in patient receiving platinum fluorouracil plus cetuximab versus platinum fluorouracil, there was significant improvement in the global health status/QoL score in the cetuximab arm (*P*: 0.0415) but no treatment differences in the social functioning scale. For QLQ-H&N35 (assessing symptom assessment scale used in the study by a questionare), the mean score for the cetuximab group was not significantly worse than that for the chemotherapy arm for all symptom scales at all postbaseline visits. At cycle 3, symptom score favored the cetuximab arm (symptoms included pain, swallowing, speech problems, and social eating) [47]. A study by Licitra et al., evaluating tumor EGFR gene copy number (using dual color FISH) as a predictive biomarker in EXTREME study patients, 71% of the patient was evaluated by eligibility criteria. No association of EGFR copy number with OS or PFS was found for patients treated with cetuximab plus platinum/5-FU [48].

#### 5.1.3. Other Monoclonal Abs to EGFR

Panitumumab is a fully human IgG2 monoclonal antibody against EGFR that has received FDA approval in colorectal cancer. In the SPECTRUM phase III trial, 657 patients were treated with cisplatin plus 5-fluorouracil, with or without panitumumab. There was unfortunately a statistically nonsignificant trend toward increase in overall survival with the addition of panitumumab (median 11.1 versus 9.0 months, HR 0.87, 95% CI 0.73–1.05) [49]. Panitumumab was also used in combination with carboplatin, paclitaxel and radiation therapy with encouraging results. In a phase I trial by Wirth et al, paclitaxel was used in 2 doses with one as 15 mg/m^2^ in 3/19 patients and 30 mg/m^2^ in 16/19 patients who are all stage III to IVB SCCHN added to panitumumab, carboplatin, and intensity-modulated radiotherapy. All patients had PR. The overall complete clinical response rate was 95% which is encouraging. At median followup of 21 months, 18 of 19 patients (95%) remained disease free. Further studies with same combination in advanced phases of clinical trials will need to define toxicity profile and overall survival with a larger cohort of patients [50]. A study with panitumumab monotherapy (Panitumumab Regimen in Second-line Monotherapy of Head and Neck Cancer (PRISM) trial; NCT00446446) as second-line therapy as well as a phase II randomized study with cisplatin and docetaxel with or without panitumumab (Panitumumab Added to Regimen for Treatment of Head and Neck Cancer Evaluation of Response (PARTNER) trial; NCT00454779) as first-line therapy of recurrent or metastatic SCCHN is currently ongoing.

Zalutumumab is another monoclonal antibody that targets the EGFR. In a randomized, phase III trial (ZALUTE), 286 patients who had progressed on platinum-based chemotherapy were randomly assigned in a 2 : 1 ratio to zalutumumab or best supportive care. There was a statistically nonsignificant increase in overall survival with zalutumumab compared with best supportive care (6.7 versus 5.2 months, hazard ratio 0.77, 95% CI 0.57–1.05). Although the primary end point, OS, was not significantly different between arms, patients treated with zalutumumab had better PFS and disease control rate. The percentage of patients surviving at 12 months was longer with zalutumumab (22 versus 12 percent) [52].

### 5.2. EGFR Tyrosine Kinase Inhibitors

Gefitinib and erlotinib are well studied as selective EGFR TKIs and have been extensively investigated in SCCHN.

Erlotinib is an orally available, potent, reversible, and selective inhibitor of the EGFR tyrosine kinase. In a phase I/II trial to determine the dose and toxicity of erlotinib in HNSCC patients with no prior chemotherapy and measurable disease who were treated in three escalating-dose cohorts of daily continuous oral erlotinib and intermittent intravenous (IV), cisplatin given every 21 days showed favorable toxicity profile and has antitumor activity in HNSCC comparable to standard combination chemotherapy regimens. 51 patients were enrolled, 44 and 43 were eligible for toxicity and efficacy evaluations, respectively. The intention-to-treat response rate was 21%, with one complete and eight partial responses (95% CI, 10% to 36%), and disease stabilization was achieved in 21 patients (49%; 95% CI, 33% to 65%). Median progression-free survival was 3.3 months (95% CI, 2.7 to 4.8 months) and median overall survival was 7.9 (95% CI, 5.6 to 9.5) months. The combination was well tolerated, with minimal grade 3 or higher toxicity. The most frequent grade 1 to 2 toxicities encountered, based on percentage of cycles delivered, were rash (68%), hypomagnesemia (51%), anemia (29%), fatigue (23%), lymphopenia (23%), and dry skin (21%). Adverse events of grade 3 or worse were rare; the most frequent were fatigue and lymphopenia, seen in 3% of treatment cycles [56]. In another phase II trial where patients with locally recurrent and/or metastatic HNSCC were treated with erlotinib, one-hundred fifteen patients were enrolled. Disease stabilization was maintained in 44 patients (38.3%) for a median duration of 16.1 weeks. The median progression-free survival was 9.6 weeks (95% CI, 8.1 to 12.1 weeks), and the median overall survival was 6.0 months (95% CI, 4.8 to 7.0 months). Subgroup analyses revealed a significant difference in overall survival favoring patients who developed at least grade 2 skin rashes versus those who did not (*P*: 0.045), whereas no difference was detected based on HER1/EGFR expression. Rash and diarrhea were the most common drug-related toxicities, encountered in 79% and 37% of patients, respectively, though the severity was mild to moderate in most cases. In this trial using single-agent erlotinib reported an ORR of 4%, median PFS of 2.2 months, and OS of 6 months [59].

In a study combining Erlotinib and Bevacizumab in recurrent metastatic SCCHN, 48 patients were selected with no more than 1 line of prior therapy to be treated with Bevacizumab 15 mg/kg q3wk and erlotinib 150 mg with the primary endpoint in phase II being response rate and PFS. Seven patients had a response, with four showing a complete response allowing rejection of the null hypothesis. Median time of overall survival and PFS was 7.1 (95% CI 5.7–9.0) and 4.1 months (2.8–4.4), respectively [57]. In a phase II trial utilizing Iressa (ZD1839) 52 patients with recurrent metastatic SCCHN were enrolled. Half the cohort received ZD1839 as second-line therapy. Forty-seven patients were assessable. A response of 10.6% was reported with a disease control rate of 53%. Median time to progression and survival was 3.4 and 8.1 months, respectively. Three patients had grade 3 diarrhea, performance status and development of skin toxicity were found to be strong predictors of response, progression, and survival [58].

A phase III trial (IMEX) conducted by Stewart et al. comparing Methotrexate alone to Gefitinib alone in recurrent head and neck cancers failed to show a prolonged median overall survival compared to weekly Methotrexate. In this trial, 2 doses of Gefitinib were used at 250 mg and 500 mg daily compared to 40 mg/m2 weekly intravenous infusion of Methotrexate, and both of these doses failed to show an improvement in survival [53]. Gefitinib 250 mg/day in combination with docetaxel 35/m2 on days 1, 8, and 15 of a 4-week cycle was compared to Docetaxel alone in a phase III trial of patients with recurrent metastatic SCCHN with performance status of 2 who received or did not receive chemotherapy. The study was terminated at interim analysis in November 2008 because it was highly unlikely that the primary endpoint could be met. The docetaxel/gefitinib combination improved median time to tumor progression (3.5 versus 2 months, P: 0.047), but no response rate (12% versus 6%, *P* = 0.21), progression-free survival (3.3 versus 2.2 months, P: 0.18) are or overall survival (6.8 versus 6.2 months, P: 0.97), compared with docetaxel. Overall, the results did not favor the experimental arm. One criticism of this study is allowing patients who received multiple lines of chemotherapy with relatively poor performance status to be included in addition to the 250 mg dose of gefitinib which is of questionable effectiveness [54].

Lapatinib is an orally active drug for breast cancer and other solid tumors. It is a dual tyrosine kinase inhibitor which interrupts the HER2 growth receptor pathway [60]. As a single agent in recurrent/metastatic SCCHN has little activity in either EGFR inhibitor naïve or refractory subjects. A phase II study enrolled 107 therapy-naïve patients with locally advanced SCCHN randomized (2 : 1) to receive lapatinib or placebo for 2–6 weeks before chemoradiation therapy (CRT), aimed at studying the apoptosis rate, toxicity and clinical response in the subset of patients. The authors concluded no effect of lapatinib on, apoptosis rate. Heterodimerization of EGFR with other ErbB/HER receptors is important for EGFR signaling pathway activation and may contribute to resistance to EGFR inhibition. Dual or pan-HER inhibitors can potentially overcome resistance by this mechanism. BIBW 2992 (afatinib) is a novel, orally bioavailable irreversible inhibitor of EGFR and HER2 receptor tyrosine kinases. Preclinical data showed that BIBW 2992 displays potent activity against multiple EGFR mutations. It is a potent, orally bioavailable irreversible inhibitor of EGFR/HER1 and HER2 receptor tyrosine kinases. Patients with metastatic or recurrent SCCHN after failure of platinum-containing therapy were randomized to receive 50 mg of BIBW 2992 daily or weekly, cetuximab 400 mg/m2 (loading), and 250 mg/m2 thereafter until disease progression or undue side effects (stage 1), with a crossover design after disease progression (stage 2). 124 patients were randomized; 15/124 patients were not evaluable for tumor response evaluation. Of the 109 patients evaluable, 35 have not yet undergone postrandomization tumor imaging. Among 34/74 patients randomized to BIBW 2992, 6/34 (18%) patients showed a partial response (PR), 18/34 (53%) patients revealed stable disease (SD) and 10/34 (30%) patients showed progressive disease (PD). Among the 40/74 patients randomly assigned to cetuximab, there were 3/40 (8%) patients with PR as best response. 20/40 (50%) patients with SD and 17/40 (43%) patients with PD. Preliminary safety analyses revealed side-effect profiles typical for EGFR inhibitors, with diarrhea and skin-related adverse events. Preliminary efficacy analysis based upon response rate suggests that BIBW 2992 is active in patients with metastatic or recurrent SCCHN after failure of platinum-containing therapy and compares favorably to patients receiving cetuximab [55]. Additional studies in the recurrent metastatic setting as well as post therapy maintenance setting are currently underway.  Table 1 is a summary of trials involving EGFR inhibitors in the treatment of recurrent or metastatic head and neck squamous cell carcinoma.

## 6. Resistance to EGFR-Targeted Therapy

Resistance to EGFR-targeted therapy is mediated through alternate means of extracellular signal-regulated kinase 1/2 (ERK1/2) activation that bypasses EGFR either via alternative receptors at the plasma membrane or constitutively active downstream components. By generating cetuximab-resistant cell lines, Yonesaka et al. first identified multiple clones that exhibited less effective suppression of ERK1/2 phosphorylation in the presence of cetuximab. Further analysis of these clones revealed amplification of ERBB2 with corresponding increases in total and phospho-*ERBB2* levels. Subsequent depletion of ERBB2 in the resistant clones restored sensitivity to cetuximab, confirming the importance of ERBB2 in the resistant phenotype. ERBB2 amplification is the proposed mechanism of cetuximab-resistant clones where acquired resistance was mediated by increased levels of heregulin, a ligand that binds ERBB3 and ERBB4. This leads to activation of downstream pathway targets and the role of this ligand is yet to be defined [61]. Similar to cetuximab resistance which is overcome through bypass signaling, other EGFR-targeted agents were studied. In nonsmall cell lung cancer, amplification of *MET* is associated with resistance to the reversible EGFR TKI gefitinib via ERBB3 activation [62]. As part of EGFR variants, the III variant (EGFRvIII) was identified as the most commonly altered one with a truncated ligand which is the result of a mutation that eliminates exons 2–7 resulting in a distorted ligand-binding region [63]. This variant is expressed on 40% of SCCHN tumors and is responsible for increased proliferation, tumor growth, and chemotherapy resistance to antitumor drugs including the EGFR targeting moAb cetuximab [3]. The activation of EGFRvIII was demonstrated to induce invasion by its effect on increasing the STAT 3 activation pathway. Cetuximab effect in inhibiting this pathway was shown to be in tumors expressing wild-type EGFR and not EGFRvIII, thus proving that expression of EGFRvIII on tumor cells might result in resistance to cetuximab [64]. EGFRvIII decreased SCCHN cell apoptosis in response to cisplatin and decreased growth inhibition following treatment with cetuximab [65].

## 7. Targeted Agents beyond EGFR

Insulin growth factor-1 (IGF-1) receptor (IGF1R) generates potent prosurvival signals and has been implicated in therapeutic resistance; its ability to induce resistance to EGFR-TKIs was studied in vitro. Five HNSCC cell lines showed reduced sensitivity to the EGFR-TKI gefitinib when the IGF1R was activated. In this study, it was shown that IGF1R activation blocks the apoptotic potential of the cell [66]. This is supported by Bohula et al. in their experiments which proved that IGF-1 and IGF-2 are ubiquitously produced protein hormones that interact with the IGF-1 receptor (IGF1R) to regulate growth, differentiation, and survival. The IGF1R activates both Ras/Erk- and PI3K/Akt-related signal transduction pathways, which act to promote proliferation and prevent apoptosis [67]. Recent results of IGF inhibitors in SCCHN have not shown promise. Future studies are focusing on combining EGFR inhibitors with other targeted agents with possible synergistic effects. Studies at several centers including ours are examining these combinations with other targeted agents to the mTOR, COX-2, and other pathways.

## 8. Conclusion

EGFR inhibitors have become an established part of SCCHN treatment. These agents are used in metastatic and concurrent setting with a noted clinically significant benefit. Novel therapies targeting pathways downstream of EGFR are used to circumvent possible mechanisms of resistance to EGFR targeted therapies. Ongoing studies are combining mTOR inhibitors and angiogenesis inhibitors to EGFR inhibitors as second-/third-line treatment to overcome the resistance. To this date, cetuximab is the only targeted agent that produced OS benefit in a phase III randomized trial in recurrent or metastatic SCCHN and when used for locally advanced disease concurrent with radiation therapy. The role of other EGFR monoclonal antibodies or TKIs is yet to be better defined SCCHN. Strategies of simultaneous targeting two or more signaling pathways, such as, VEGFR or EGFR, and IGF1R or target downstream of EGFR such as mTOR, P13K/AKT, are under investigation.

## Figures and Tables

**Table 1:** Summary of EGFR inhibitors in recurrent or metastatic head and neck squamous cell carcinoma.

Trial	Line of treatment	Phase	No. of patients	Medication	Overall response	Median OS (mo)	Median PFS (mo)
Monoclonal antibodies							

E5397 Burtness et al. [45]	First	III	117	Cisplatin versus cisplatin/cetuximab	10% versus 26% (*P* 0.03)	8 versus 9.2 (*P* 0.21)	2.7 versus 4.2 (*P* 0.09)
Vermorken et al. [39]	Second (platinum refractory)	II	103	Cetuximab	13%	6	
EXTREME Vermorken and Specenier [51]	First	III	442	Platinum-based versus platin + cetuximab	20% versus 36% (*P* < 0.01)	7.4 versus 10.1 (*P* 0.04)	3.3 versus 5.6 (*P* < 0.001)
SPECTRUM Vermorken et al. [49]	First	III	657	Cis/5FU versus Cis/5FU + panitumumab	25% versus 36%	9 versus 11.1 (*P* 0.14)	4.6 versus 5.8 (*P* 0.004)
Zalute Machiels et al. [52]	Second after platinum	III	286	Zalutumumab versus support or methotrexate	1.1 and versus 1.1%	6.7 versus 5.2 (*P* 0.065)	9.9 versus 8.4 (*P* 0.001)
Herbst et al. [42]	Second	II	132	Cis/cetuximab after progression on cis	26%	6.1 and 4.3	
Hitt et al. [40]	Second	II	46	Paclitaxel + cetuximab	60%		5.6
Baselga et al. [44]	Second	II	96	Cetuximab + platinum	10%	6.1	2.8

Tyrosine kinase inhibitors							

IMEX Stewart et al. [53]	Second after platinum/no platinum	III	486	Gefitinib 250 versus gefitinib 500 versus methotrexate	2.7% versus 7.6% versus 3.9%	5.6 versus 6 versus 6.7	
E1302 Argiris et al. [54]	Any	III	270	Docetaxel versus docetaxel + gefitinib 250	6% versus 12% (*P* 0.21)	6.18 versus 6.83 (*P* 0.97)	2.2 versus 3.35 (*P* 0.18)
BIBW2992 Seiwert et al. [55]	After platinum	III	124	Afatinib versus cetuximab	22% versus 13%		3.75 versus 2.35
Siu et al. [56]	First	I/II	51	Erlotinib + cis	21%	7.9	3.3
Cohen et al. [57]	Second	I/II	48	Erlotinib + bevacizumab	14.5%	7.1	4.1
Cohen et al. [58]	First and second	II	52	Gefitinib	10.6%	8.1	3.4
